# Supplementary material for: Assessing the performance of Moghani crossbred lambs derived from different mating systems with Texel and Booroola sheep
Source: PLoS One. 2024 Apr 4;19(4):e0301629. doi: 10.1371/journal.pone.0301629 (PMC10994311; doi:10.1371/journal.pone.0301629)
Supplement: S1 Fig — (PDF) [file pone.0301629.s001.pdf]

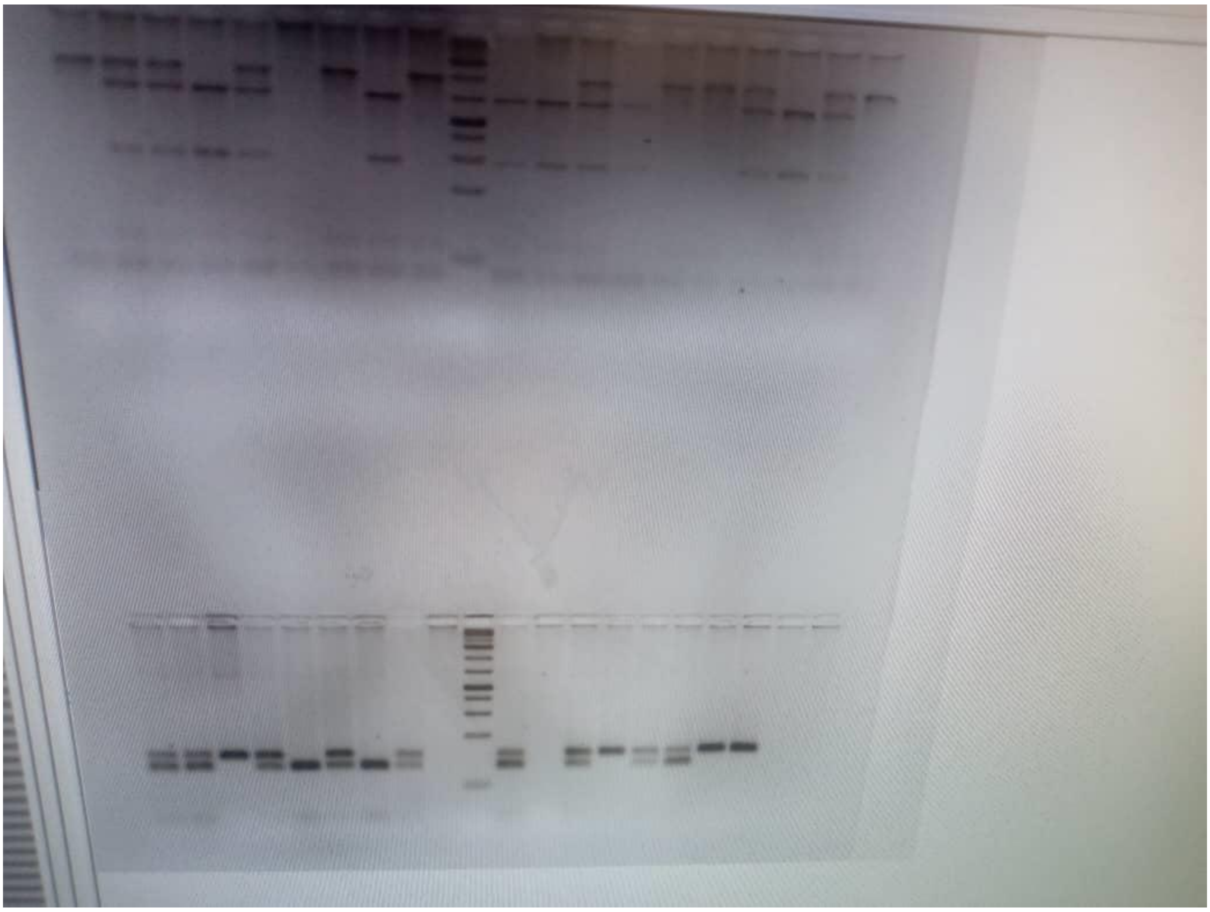

S1 Fig. Original image depicting PCR-RFLP band patterns migrated on a 3.5% agarose gel through electrophoresis.
