# Supplementary material for: Assessing the performance of Moghani crossbred lambs derived from different mating systems with Texel and Booroola sheep
Source: PLoS One. 2024 Apr 4;19(4):e0301629. doi: 10.1371/journal.pone.0301629 (PMC10994311; doi:10.1371/journal.pone.0301629)
Supplement: Attachment — Submitted filename: Manuscript_PLOS ONE 22-1-2024.docx [file pone.0301629.s002.docx]

Assessing the performance of Moghani crossbred lambs derived from different mating systems with Texel and Booroola sheep

Reza Talebi^1,*^, Mohsen Mardi^1^, Mehrshad Zeinalabedini^1^, Mehrbano Kazemi Alamouti^1^, Stéphane Fabre^2^, Mohammad Reza Ghaffari^1,*^

^1^ Department of Systems and Synthetic Biology, Agricultural Biotechnology Research Institute of Iran, Agricultural Research, Education and Extension Organization (AREEO), Karaj, Iran

^2^ GenPhySE, Université de Toulouse, INRAE, ENVT, 31326 Castanet Tolosan, France

0000-0002-5555-8453 (R. Talebi)

0000-0002-3436-4334 (M. Zeinalabedini)

0000-0001-7350-9500 (S. Fabre)

0000-0002-7139-8613 (M.R. Ghaffari)

* Corresponding authors:

[talere1986@gmail.com](mailto:talere1986@gmail.com) (R. Talebi),

[mrghaffari52@gmail.com](mailto:mrghaffari52@gmail.com) (M.R. Ghaffari)

**Abstract**

In our ongoing project, which focuses on the introgression of *Booroola*/*FecB* gene and the *myostatin* (*MSTN*) gene into purebred Moghani sheep, we assessed the performance of second-generation Moghani crossbreds such as second crossbreds (F2) and initial backcross generation (BC1). These crossbreds were generated through different mating systems, including in-breeding, outcrossing, first paternal backcrossing (PBC1), and first maternal backcrossing (MBC1). Notably, F2 strains exhibited lean tail, woolly fleece and a higher percentage of white coat color compared to BC1. The impact of mating systems and birth types on pre-weaning survival rates was found to be statistically significant (*P* < 0.0001), with singleton offspring resulting from paternal backcross showing a particularly substantial effect. The F2 crossbred lambs carrying the *Booroola* gene exhibited significantly enhanced survivability compared to those carrying the *MSTN* gene, suggesting a positive influence of the *Booroola* prolificacy gene on survival outcomes. However, the occurrence of multiple births has a significant negative impact on lamb survival (*P* < 0.0001). The PBC1 sheep strains, specifically Texel Tamlet ram strains carrying the *MSTN* mutation, exhibited superior growth rates compared to others (*P* < 0.05). Interestingly, the *MSTN* mutation in the homozygous variant genotype significantly impacts growth rate before weaning compared to other genotypes and pure Moghani sheep (*P* < 0.05). The findings underscore the importance of considering genetic factors in sheep breeding programs to enhance growth until weaning, crucial for sheep fattening and ewe reproduction management.

**Keywords**

Gene introgression; Growth traits; Mating systems; Morphometric characteristics; Sheep

**Introduction**

Mating systems play a crucial role in influencing the performance and genetic traits of livestock. Researchers have investigated a range of mating systems, including pure-breeding, crossbreeding, outcrossing, and backcrossing, to evaluate their effects on vital characteristics such as growth traits, lamb survival, and ewe prolificacy [1–6]. Farm animals' growth rate significantly impacts profitability [7]. Crossbreeding with major gene carriers, using local or exotic germplasm, and genomic introgression offer promising pathways to achieve substantial genetic gains [8]. Successful sheep crossbreeding programs often involve various mating systems, such as F1 crosses, three-way crosses, and composite breeds [9]. This approach accelerates genetic progress, overcoming challenges associated with direct selection for quantitative traits, leading to more productive and profitable sheep farming operations.

Mature body size in sheep is known to be influenced by a higher degree of polygenic factors when compared to other domesticated species [10]. This means that many genes, each with a small effect, contribute to mature body size. This complex genetic nature presents a significant challenge, rendering classical breeding methods alone insufficient in achieving desired outcomes [11]. Consequently, the implementation of genomic selection programs becomes essential to effectively address this complexity and optimize breeding efforts. Genomic selection is a breeding method that uses information from an animal's genome to predict its genetic merit for a desired trait. This information can be used to select animals for breeding that are more likely to produce offspring with the desired trait [12,13].

Numerous indigenous sheep breeds lack key causative mutations linked to desirable traits like muscle hypertrophy and prolificacy. Introducing breeds carrying major genes enables substantial improvements in these desirable traits. Efforts to enhance these attributes involve crossbreeding initiatives, aiming to introduce major genetic factors. As reviewed by Talebi et al. [14] emphasized the significance of the OAR2 region, which houses the *myostatin* (*MSTN* g+6223G>A) gene responsible for double muscling in various sheep breeds, including New Zealand Texel [15,16], Australian Texel [17], Belgian Texel [18,19], Norwegian White Sheep [20] and the commercial Charollais sheep [21]. For instance, the Texel sheep breed's *MSTN* g+6223G>A has been successfully introduced into Ramlıç sheep breeds [22,23]. Similarly, the highly prolific *Booroola*/*FecB* allele of the *bone morphogenetic protein receptor type 1B* (*BMPR1B*) gene, initially identified in the Booroola Merino breed, has been effectively incorporated into diverse sheep breeds [24]. The *FecB* allele has resulted in sheep with increased litter size and prolificacy. Noteworthy instances include its successful integration into Afshari breed [25,26], Assaf [27,28], Avassi [27–29], Deccani [30], Mérinos d’Arles [31], Moghani [32,33], and Rambouillet [34]. These examples illustrate the potential of major genes introgression to improve the genetic merit of sheep breeds.

Iran's 27 indigenous sheep breeds have the potential to play a crucial role in meeting future global protein demands and becoming essential contributors to the export market. However, many of these breeds exhibit low prolificacy, slow growth rates, inefficient feed conversion, and yield carcasses with poor composition [11,35–38]. The Moghani sheep is a local breed in Iran that is raised primarily for meat production. It is known for its large fat tail and lower prolificacy, but it also has the advantage of being able to reproduce out of season [5]. However, the Moghani sheep faces challenges in terms of economic profitability. Numerous studies have shown that the genetic progress for growth and reproductive traits in this breed is slow, due to low heritability estimates [39–42]. To sustain and enhance the value of the Moghani sheep in the sheep industry, it is important to address these challenges and focus on selective breeding.

As part of a national initiative, we launched an introgression project to enhance the productivity of Moghani sheep. This was achieved through strategic crossbreeding with high-yielding sheep breeds, specifically Texel and Booroola sheep. These breeds possess crucial genetic factors known for enhancing traits such as muscularity and prolificacy. The comprehensive introductory details and outcomes of the F1 crosses, including Booroola Merino×Moghani (BMM), Booroola Romney×Moghani (BRM), Texel Tamlet×Moghani (TTM), and Texel Dalzell×Moghani (TDM), have been extensively documented in our prior publications [4,5]. In our ongoing project, we bred second-generation Moghani crossbreds using various mating systems, including in-breeding, outcrossing, and backcrossing. This study specifically focuses on a comprehensive comparison of growth performance, fat-tail traits, and lamb coat colors between purebred Moghani sheep and second-generation crossbreds. Additionally, we investigated the impact of distinct genotypes of introgressed genes, particularly prolificacy *Booroola*/*FecB* and hyper-muscularity *myostatin* (*MSTN* g+6223G>A), on the performance of these second-generation Moghani crossbreds. It's important to note that, despite our meticulous examination of these traits, we unfortunately couldn't evaluate prolificacy due to the lack of available records. Acknowledging the significance of this parameter, we plan to include it in future investigations as more data becomes accessible.

**Materials and methods**

**Ethics statement**

The data collection formats and procedures employed in this study underwent thorough review and approval by the Animal Care and Use Committee at the Agricultural Biotechnology Research Institute (ABRII) in Karaj, Alborz, Iran. The committee granted approval for all procedures and activities involving animals, ensuring strict adherence to local guidelines. The study exclusively relied on data obtained from live sheep at the breeding facility of Jovain Agricultural & Industrial Corporation in Jovain, Razavi Khorasan, Iran. It is important to note that no invasive procedures were conducted, and the animals were closely monitored by researchers. The study did not involve anesthesia, euthanasia, or animal sacrifice.

**Management of housing conditions, feeding regimens, and health monitoring**

The animals were raised at the breeding facility of Jovain Agricultural & Industrial Corporation in Jovain, Razavi Khorasan, Iran (Jovain, Razavi Khorasan, Iran. Latitude: 36.655297/N 36° 39' 19.06800″, Longitude: 57.423406/57° 25' 24.26100″). Jovain County experiences an average annual rainfall of approximately 272 mm, with a mean daily temperature range of 17.8 to 29.5˚C, characterizing it as a moderately warm climate zone. A semi-intensive management system, characterized by a moderate amount of production inputs, was employed for animal care.

The animals were permitted to graze or browse on natural pasture for approximately six hours during the daytime. Additionally, they received a supplementary diet of 0.10 to 0.40 kg concentrate mixture per day, consisting of alfalfa barn, maize silage, and salt. The amount varied based on factors such as age, physiology, and sex.

Housing arrangements were organized according to sex, physiological status, and health status. Animals had access to water *ad libitum* and were subjected to vaccinations against prevalent diseases in the area. Regular treatments, deworming, and scheduled spraying were conducted to maintain their health. Each kid was assigned a unique identifying number, and their birth weight was recorded within 24 hours of birth. Kids were kept indoors with their dams for three to seven days, after which dams were moved outdoors, and kids were allowed to suckle three times a day until reaching the weaning age of 90 days. Animal care staff performed routine health assessments to ensure the overall well-being of the animals.

**Crossbreeding to produce first generation progenies**

As previously detailed in [5], a total of 380 Moghani pure sheep (3-year-old ewes) underwent artificial insemination in September 2019. Frozen sperm from two New Zealand Booroola rams (one from the Merino strain and one from the Romney strain), both homozygous carriers (GG) of the *Booroola*/*FecB* mutation (OAR6:g.29382188A>G; NC_019463.1, Oar_v3.1, rs418841713), and from two New Zealand Texel rams (one from the Dalzell strain and one from the Tamlet strain), both homozygous carriers (AA) of the *MSTN* g+6223G>A mutation (OAR2:g.118150665G>A; NC_019459.1, Oar_v3.1, rs408469734), were used in the insemination process. The performance of the first generation of crossbred lambs (F1 crosses), including Booroola Merino×Moghani (BMM), Booroola Romney×Moghani (BRM), Texel Tamlet×Moghani (TTM), and Texel Dalzell×Moghani (TDM), was described in [5].

**Mating systems to produce second generation progenies**

To produce second-generation crossbreds including second crossbreds (F2) and initial backcross generation (BC1), we used mating systems including in-breeding, outcrossing, backcrossing, and pure-breeding. The design of the mating systems is shown in Fig 1. All systems were carried out using synchronized ewes with controlled internal drug release (CIDR) devices. In the in-breeding process, the F1 crosses were mated in half-sib states, e.g., TTM×TTM (TTMTTM-2), TDM×TDM (TDMTDM-2), BRM×BRM (BRMBRM-2) and BMM×BMM (BMMBMM-2). Therefore, the progenies had at least 12.5% inbreeding coefficient. In the outcrossing system, various strains of F1 crosses were mated while the sire and dam were not related, e.g., TDM×TTM (TDMTTM-2), TTM×TDM (TTMTDM-2). The initial paternal backcross generation (PBC1) was established between four types of F1 crossbred rams and purebred Moghani ewes: BMM×Moghani (BMMM-2), BRM×Moghani (BRMM-2), TDM×Moghani (TDMM-2), and TTM×Moghani (TTMM-2). The initial maternal backcross generation (MBC1) was established between purebred Moghani rams (3 years old) and two types of F1 crossbred Booroola ewes: Moghani×BRM (MBRM-2) and Moghani×BMM (MBMM-2).

**Fig 1. Mating systems utilized to produce crossbred lambs. A.** It depicts F1 crosses including Texel Tamlet × Moghani (TTM), Texel Dalzell × Moghani (TDM), Booroola Merino × Moghani (BMM) and Booroola Romney × Moghani (BRM). **B.** It depicts pure-breeding system to produce purebred Moghani (M) lambs. **C.** It depicts in-breeding system to produce inbred F2 lambs from half-sibs, Texel strains (TTMTTM-2, TDMTDM-2) and Booroola strains (BMMBMM-2, BRMBRM-2). **D.** It depicts outcrossing system to produce F2 lambs from non-relative parents, Texel strains (TTMTDM-2, TDMTTM-2). **E.** It depicts paternal backcrossing system to produce first generation of paternal backcross (PBC1) lambs, Texel strains (TDMM-2, TTMM-2) and Booroola strains (BMMM-2, BRMM-2). **F.** It depicts maternal backcrossing system to produce first generation of maternal backcross (MBC1) lambs, Booroola strains (MBRM-2, MBMM-2).

**Lambing information**

In the context of in-breeding, a total of 25 lambs were born alive within the first week of birth, derived from a group of 39 lambs born to 25 uniparous F1 ewes aged 1 year. This group encompassed 14 F2 Booroola lambs (BRMBRM-2 and BMMBMM-2) as well as 15 F2 Texel lambs (TTMTTM-2 and TDMTDM-2). Turning to the subject of outcrossing, 40 live lambs were delivered among 48 offspring from 31 multiparous F1 ewes aged 2 years. Among these were F2 Texel lambs, exemplified by TDMTTM-2 and TTMTDM-2. In the case of PBC1, there was a count of 435 live lambs born from a pool of 438 lambs, all from 385 multiparous purebred Moghani ewes aged 3 years. The lambs were identified as TDMM-2, TTMM-2, BMMM-2, and BRMM-2. Shifting focus to MBC1, 34 live lambs were welcomed into the world among 36 offspring from 28 multiparous BMM and BRM ewes aged 2 years, showcasing the presence of MBRM-2 and MBMM-2 lambs.

**Records and data management**

The growth performance of purebred Moghani, and second (F2 and BC1) generations of crossbred lambs were evaluated. Before the analysis, we adjusted weights to corresponding 90 (Adjusted.W3), and 180 (Adjusted.W6) days, using the formulas as follows:

$$Adjusted.W3\left( kg \right)=\frac{90\left( W3-BW \right)}{Date.W3.BW}+BW$$

$$Diff.W3.BW\left( kg \right)=Adjusted.W3-BW$$

$$Adjusted.W6\left( kg \right)=\frac{180\left( W6-W3 \right)}{Date.W6.W3}+Adjusted.W3$$

$$Diff.W6.W3\left( kg \right)=Adjusted.W6-Adjusted.W3$$

$$ADWG.BW.W3\left( gr \right)=\frac{Diff.W3.BW\left( kg \right)}{90}\times1000$$

$$ADWG.W3.W6 \left( gr \right)=\frac{Diff.W6.W3\left( kg \right)}{90}\times1000$$

in this context, BW represents birth weight, W3 signifies the weight at 3 months of age, and W6 indicates the weight at 6 months of age. Date.W3.BW represents the duration in days from birth to 3 months, while Date.W6.W3 represents the duration in days from 3 to 6 months. The disparities between two consecutive weighings (Diff) and various average daily weight gains (ADWG) were computed, such as Diff.W3.BW for the difference in weights from birth to 3 months, Diff.W6.W3 for the difference in weights from 3 to 6 months, ADWG.BW.W3 for the average daily weight gain from birth to 3 months, and ADWG.W3.W6 for the average daily weight gain from 3 to 6 months. Additionally, fat-tail traits at 6 months of age were documented, encompassing tail/fat tail type, fat-tail height (FTH, in centimeters), and fat-tail width (FTW, in centimeters). Furthermore, morphometric of the lamb coat colors were collected in five coat colors types including white, brown, light-brown, strong brown, and black.

**Blood sample collection**

Blood samples were collected using anticoagulant blood collection tubes containing K2-EDTA to minimize stress and discomfort to the animals.

**Extraction of DNA and genotyping through PCR-RFLP method**

The extraction of genomic DNA from whole blood samples was carried out following the procedure previously described by Talebi et al. [43]. The PCR-RFLP genotyping procedures for *BMPR1B* mutation OAR6:g.29382188A>G and the *MSTN* mutation OAR2:g.118150665G>A were conducted following the methods outlined in [5].

**Statistical analysis**

The frequency of colors, mating systems, sex, introgressed gene, and type of birth were compared based on two-way chi-square tests with a significance level of 5%, using the PROC FREQ procedure in SAS Version 8.2 (2001) .

The analysis of growth traits utilized the general linear model (GLM) procedure implemented in SAS. The determination of significance (*P* < 0.05) was conducted through Duncan's multiple range test. The analysis employed the following multivariate models:

*y_ijklmnopqr_* = µ + *B_j_* + *D_k_* + *C_l_* + *T_m_* + *S_n_* + *M_o_* + *G_p_* + *L_q_* + (*B*×*D*) *_jk_* or (*C*)*_l_* + (*C*×*G*)*_lq_* (*F*×*G*)*_oq_* + (*M*×*G*)*_pq_* + *e_ijklmnopqr_*

where: *y_ijklmnopq_* is the vector of observation of the *i*^th^ animal within the *j*^th^ sire breed, *k*^th^ dam breed, *l*^th^ sheep strain (*j*^th^ sire breed× *k*^th^ dam breed), *m*^th^ type of birth, *n* sex category, *o*^th^ mating system, *p*^th^ genotype category, and *q*^th^ tail type.

μ is the overall mean, *B_j_* is the effect of the *j*^th^ sire breed (*j* = Moghani, TTM, TDM, BMM, BRM), *D_k_* is the effect of the *k*^th^ dam breed (*k* = Moghani, TTM, TDM, BMM, BRM), *C_l_* is the effect of the *l*^th^ sheep strain (*l* = Moghani, BRMBRM-2, BMMBMM-2, BMMM-2, BRMM-2, MBRM-2, MBMM-2, TTMTTM-2, TDMTDM-2, TDMTTM-2, TTMTDM-2, TDMM-2, TTMM-2), *T_m_* is the effect of *m*^th^ type of birth (*m* = 1, 2, 3, 4), *S_n_* is the effect of *n*^th^ sex (*n* = male and female), *M_o_* is the effect of *o*^th^ mating system (*o* = in-breeding, outcrossing, paternal backcrossing, maternal backcrossing, and pure-breeding), *G_p_* is the effect of *p*^th^ genotype (GG, GA, AA for *MSTN* mutation and AA, AG, GG for *BMPR1B* mutation), *L_q_* is the effect of *q*^th^ tail type (*q* = tail or fat-tailed), (*B*×*D*) *_jk_* is the interaction effect between *j*^th^ sire breed and *k*^th^ dam, (*C*×*G*)*_lp_* is the interaction effect between *l*^th^ strain and *p*^th^ genotype, (*M*×*G*)*_op_* is the interaction effect between *o*^th^ mating system and *p*^th^ genotype.

**Results and discussion**

This investigation builds upon our prior studies [4,5] regarding the introgression of the prolificacy *Booroola*/*FecB* mutation (OAR6:g.29382188A>G; NC_019463.1, Oar_v3.1, rs418841713) and the hyper-muscularity *MSTN* g+6223G>A mutation (OAR2:g.118150665G>A; NC_019459.1, Oar_v3.1, rs408469734) into purebred Moghani sheep. The objective is to develop second-generation Moghani crossbreds using various mating systems, such as inbreeding, outcrossing, and backcrossing. We conducted a systematic comparison of growth traits, fat-tail characteristics, and morphometrics of lamb coat colors between purebred Moghani sheep and their second generations (F2 and BC1) of crossbred lambs. Additionally, we evaluated the effects of each genotype (homozygous reference, heterozygous, and homozygous variant) of the *BMPR1B* and *MSTN* mutations on the body weights, growth traits, fat-tail traits, and morphometrics of lamb coat colors in second-generation crossbred lambs

**RFLP genotyping results**

The RFLP genotyping analysis of the *MSTN* mutation (OAR_v.3.1; Chr 2: g.118150665G>A) provided confirmation regarding the genotypes of F2 crossbred lambs with Texel. These genotypes included homozygous reference G/G, heterozygous G/A, and homozygous variant A/A (Fig 2A). Furthermore, the RFLP genotyping of the *BMPR1B*/*Booroola* mutation (OAR_v.3.1; Chr 6: g.29382188A>G) revealed the genotypic variations within F2 crossbred lambs with Booroola Merino and Booroola Romney. The genotypes encompassed homozygous reference A/A, heterozygous A/G, and homozygous variant G/G at this specific locus (Fig 2B). It is noteworthy that lambs with the homozygous variant genotype for both genes have exclusively been discovered in progenies resulting from a combination of in-breeding and outcrossing systems. A study by Daetwyler et al. [45] revealed a negative link between inbreeding rates and heritability. This is due to reduced genetic diversity caused by inbreeding. Hence, it underscores the necessity for precise breeding management to regulate these genetic variations.

**Fig 2. The electrophoretic result of PCR-RFLP band patterns migrated on agarose gel 3.5%.** SM: GeneRuler 1 kb Plus DNA Ladder #SM1331. **A.** PCR amplified *BMPR1B* gene digested by *Ava*II (5'-G/GWCC) restriction enzyme for *Booroola*/*FecB* mutation (OAR6: 29382188A>G, NC_019463.1 of genome assembly Oar_v3.1). The lanes depict *Booroola* mutation genotypes including homozygous reference (AA), heterozygous (AG) and homozygous variant (GG). **B.** PCR amplified ovine *myostatin* gene (*MSTN*) digested by *HpyCH4*IV (5'- A/CGT) restriction enzyme for *MSTN* mutation (OAR2: 118150665G>A, NC_019463.1 of genome assembly Oar_v3.1). The lanes depict *MSTN* mutation genotypes including homozygous reference (GG), heterozygous (GA) and homozygous variant (AA).

**Phenotype and morphometric characteristics**

The initial observable traits in the F1 crossbred lambs were a slender tail, a white coat, and a woolly white fleece. The purebred Moghani sheep, on the other hand, had a substantial fat-tail and a light-brown fleece that was either woolly or hairy [5]. Consistent with the results reported by Khaldari et al. [46,47], the mating of lean-tailed rams with fat-tailed ewes resulted in the birth of F1 crossbred lambs with slender tails, regardless of gender. Moving on to the second-generation of crossbred lambs, twelve strains generated through different mating systems have been shown in Fig 3. The offspring resulting from both in-breeding and outcrossing exhibited a lean tail, and a woolly fleece (Fig 4A-D). In the context of backcrossing progeny, these lambs displayed varying from short (including fat-rumped) to large fat-tails, as well as a woolly/hairy fleece (Fig 4E). The shape and size of a sheep's tail play a crucial role in their genetics and have important implications for their domestication, ability to thrive in various environments, productivity, and animal welfare [48]. At present, the fat-tail phenotype is not favorable for inclusion in breeding programs in Iran, as it presents several adverse consequences that are likely to impact aspects such as animal mobility, mating, food efficiency, and breeding expenses [5,14].

**Fig 3. Sheep strains generated through different mating systems.**

**Fig 4. Phenotype characteristic of coat color and fat-tail traits in second-generation crossbreds lambs (F2 and BC1). A.** The F2 offspring resulting from in-breeding with the homozygous variant (GG) genotype for *Booroola*/*FecB* mutation (OAR6: 29382188A>G, NC_019463.1 of genome assembly Oar_v3.1). **B.** The F2 offspring resulting from in-breeding with the homozygous variant genotype (AA) for *MSTN* mutation (OAR2: 118150665G>A, NC_019463.1 of genome assembly Oar_v3.1). **C.** The F2 offspring resulting from outcrossing with the homozygous variant genotype (AA) for *MSTN* mutation (OAR2: 118150665G>A, NC_019463.1 of genome assembly Oar_v3.1). **D.** The F2 offspring resulting from outcrossing with the homozygous variant (GG) genotype for *Booroola*/*FecB* mutation (OAR6: 29382188A>G, NC_019463.1 of genome assembly Oar_v3.1). **E.** The BC1 offspring resulting from initial backcross generation.

As indicated in Table 1, the F2 crossbred strains, including BRMBRM-2, BMMBMM-2, TTMTTM-2, TDMTDM-2, TDMTTM-2, and TTMTDM-2, exhibited a notably higher percentage of white coat color compared to the BC1 strains, which encompassed BMMM-2, BRMM-2, TDMM-2, TTMM-2, MBRM-2, and MBMM-2. Notably, all lambs born from the BMMBMM-2 strain displayed a white coat color (see Table 1). It is noteworthy that none of the parents with a black coat color were intentionally selected from the F1 crossbred and Moghani pure sheep populations to create the F2 and BC1 strains. Nonetheless, we observed the occurrence of black coat color in the sheep strains listed in Table 1, including BMMM-2 (1.02%), BRMM-2 (1.27%), MBMM-2 (5.88%), TTMTDM-2 (16.67%), TDMM-2 (2.88%), and TTMM-2 (1.52%). Given the intricate nature of the coat color trait, it has been proposed that the phenotypic variation in coat colors may be influenced by multiple genes. It was expected that various loci or genes would interact epistatically, contributing to the broad spectrum of sheep coat colors [49].

**Table 1. Percentage rate of coat color patterns across produced strains of progenies between pure Moghani sheep and second generations of crossbred lambs (F2 and BC1)**

| **Breed** | **Strains** | **N** | **Percentage rate of coat color** | | | | |
| --- | --- | --- | --- | --- | --- | --- | --- |
|  |  |  | **white** | **brown** | **light-brown** | **strong brown** | **black** |
| Booroola crossbred lambs | BRMBRM-2 | 17 | 61.11 | 0.00 | 33.33 | 5.56 | 0.00 |
|  | BMMBMM-2 | 4 | 100.00 | 0.00 | 0.00 | 0.00 | 0.00 |
|  | BMMM-2 | 96 | 33.67 | 35.71 | 29.59 | 0.00 | 1.02 |
|  | BRMM-2 | 153 | 34.18 | 37.34 | 27.22 | 0.00 | 1.27 |
|  | MBRM-2 | 15 | 6.67 | 40.00 | 53.33 | 0.00 | 0.00 |
|  | MBMM-2 | 17 | 0.00 | 41.18 | 52.94 | 0.00 | 5.88 |
| Texel crossbred lambs | TTMTTM-2 | 8 | 75.00 | 0.00 | 25.00 | 0.00 | 0.00 |
|  | TDMTDM-2 | 9 | 77.78 | 0.00 | 11.11 | 11.11 | 0.00 |
|  | TDMTTM-2 | 14 | 85.71 | 0.00 | 14.29 | 0.00 | 0.00 |
|  | TTMTDM-2 | 12 | 33.33 | 16.67 | 33.33 | 0.00 | 16.67 |
|  | TDMM-2 | 96 | 36.54 | 26.92 | 33.65 | 0.00 | 2.88 |
|  | TTMM-2 | 63 | 46.97 | 30.30 | 21.21 | 0.00 | 1.52 |
| Pure breed | Moghani | 172 | 0.00 | 0.00 | 100.00 | 0.00 | 0.00 |
| N: number of lambs at each group | | | | | | | |

**Preweaning lamb survival**

The data pertaining to preweaning lamb survival across various variables such as mating systems, sex, introgressed gene, and type of birth has been detailed in Table 2. The mating systems and type of birth significantly affected lamb’s pre-weaning survival rates (refer to Table 2, *P* < 0.0001). Notably, singleton offspring resulting from paternal backcross exhibited a substantial impact. While females exhibited a slightly improved survival rate compared to males, the observed differences lacked statistical significance (refer to Table 2, *P* > 0.05). These findings align with prior researches [6,50] that also noted fluctuations in lamb survival rates. Our study corroborates these observations and indicates that the presence of introgressed *MSTN* and *Booroola* genes does not exert a noteworthy impact on lamb survivability (see Table 2). Intriguingly, F2 crossbred lambs carrying the *Booroola* gene displayed notably enhanced survivability in comparison to lambs carrying the *MSTN* gene. The findings demonstrate that the *Booroola* prolificacy gene does not have a detrimental impact on lamb survivability. However, it is important to highlight that we observed a decline in lamb survival during cases of multiple births (refer to Table 2). This observation aligns with previous unfavorable findings when the *Booroola* mutation was introduced into Australian [51] and American [52] sheep breeds. In these studies, lamb mortality notably increased among highly prolific ewes managed in extensive conditions. This occurrence can be linked to the counteractive correlation between heightened prolificacy and lamb survival rates, combined with an increased susceptibility to pregnancy toxemia in ewes [24]. The ability of fetuses to resist hypoxia is critically significant in pregnancies with multiple fetuses, as hypoxia can have a significant impact on fetal survival and birth weight [53].

**Table 2. Preweaning survival rate (%) across mating systems, sex, introgressed gene, and type of birth in second (F2 and BC1) generations of crossbred lambs**

| **Mating systems** | Survival rate (%) | Chi.Square (*X*^2^) | *P* Value | **Sex** | Survival rate (%) | Chi.Square (*X*^2^) | *P* Value | **Introgressed gene** | Survival rate (%) | Chi.Square (*X*^2^) | *P* Value | **Type of birth** | Survival rate (%) | Chi.Square (*X*^2^) | *P* Value |
| --- | --- | --- | --- | --- | --- | --- | --- | --- | --- | --- | --- | --- | --- | --- | --- |
| in-breeding | 64.10 | 162.84 | <0.0001 | male | 93.28 | 0.261 | 0.609 | *MSTN* | 92.07 | 1.982 | 0.159 | singleton | 96.40 | 44.682 | <0.0001 |
| outcrossing | 57.14 |  |  |  |  |  |  |  |  |  |  | twins | 91.04 |  |  |
| paternal backcross | 99.31 |  |  | female | 94.33 |  |  | *Booroola* | 95.01 |  |  | triplets | 33.33 |  |  |
| maternal backcross | 94.44 |  |  |  |  |  |  |  |  |  |  | quadruplets | 26.67 |  |  |

?????????????????????????????????????????

**Effect of birth type on growth performance of lambs**

The impact of birth type on the growth performance of second generations of crossbred lambs (F2 and BC1) is presented in Table 3. Pre-weaning weights, such as birth weight (BW) and weaning weight (W.3), exhibited a significant difference. Quadruplets and triplet-born lambs displayed notably lower weights compared to twins and single-born lambs (*P* < 0.0001, see Table 3). In contrast, post-weaning, triplets exhibited a faster growth rate than single-born, twins, and quadruplet-born lambs, with an increase of 255.06 g/d (*P* < 0.9129, refer to Table 3). These findings align with the results of our prior study on F1 crossbreds [5]. A study has demonstrated that ewes rearing triplets produce 21% more milk and exhibit greater feed-to-milk conversion efficiency when compared to ewes of similar weight rearing twins [54]. This is likely attributable to the influence of the number of lambs nursed on ewe lactation. In contrast, McHugh et al. [55] found that lambs born and reared as triplets exhibited a notably slower growth rate of 299 g/d. Additionally, in a study examining the impact of the *Booroola* gene on the growth performance of Garole × Malpura sheep, Kumar et al. [56] found that the type of birth had a significant effect (*P* < 0.01) on body weight from birth to 12 months of age. Notably, single-born lambs exhibited a significantly higher body weight (*P* < 0.01) compared to twins and triplets within the same age range. Furthermore, the type of birth had a significant impact (*P* < 0.01) on the average daily weight gain before weaning, while it did not significantly affect the average daily weight gain after weaning.

**Table 3. Predicted means for the type of birth effects on lamb growth traits of pure Moghani sheep and second generations of crossbred lambs (F2 and BC1)**

| **Traits** | **Type of birth** | | | | | | | | | | | |  | SEM | *P* Value |
| --- | --- | --- | --- | --- | --- | --- | --- | --- | --- | --- | --- | --- | --- | --- | --- |
|  | **Singletons** | |  | **Twins** | |  | **Triplets** | | |  | **Quadruplets** | |  |  |  |
|  | N | Mean |  | N | Mean |  | N | Mean | |  | N | Mean |  |  |  |
| BW (kg) | 428 | 4.70 ^a^ |  | 237 | 4.11 ^a^ |  | 4 | | 2.80 ^b^ |  | 7 | 2.67 ^b^ |  | 0.23 | <0.0001 |
| W.3 (kg) or weaning | 428 | 26.67 ^a^ |  | 237 | 25.20 ^ab^ |  | 2 | | 23.20 ^ab^ |  | 7 | 22.55 ^b^ |  | 0.31 | <0.0001 |
| W.6 (kg) | 428 | 47.50 |  | 237 | 46.97 |  | 2 | | 46.14 |  | 7 | 43.01 |  | 0.48 | 0.1798 |
| Diff.W3.BW (kg) | 428 | 22.13 ^a^ |  | 237 | 21.06 ^ab^ |  | 2 | | 20.58 ^ab^ |  | 7 | 19.88 ^b^ |  | 0.19 | 0.0009 |
| Diff.W6.W3 (kg) | 428 | 20.76 |  | 237 | 21.90 |  | 2 | | 22.96 |  | 7 | 20.47 |  | 0.36 | 0.9129 |
| ADWG.BW.W3 (gr) | 428 | 245.85 ^a^ |  | 237 | 234.01 ^ab^ |  | 2 | | 228.71^ab^ |  | 7 | 220.87 ^b^ |  | 2.10 | 0.0009 |
| ADWG.W3.W6 (gr) | 428 | 230.63 |  | 237 | 243.16 |  | 2 | | 255.06 |  | 7 | 227.41 |  | 4.01 | 0.9129 |
| N: number of lambs at each group; SEM: standard error of mean; BW: birth weight; W.3: weight at 3 months (weaning); W.6: weight at 6 months; Diff.W3.BW: difference of weights at birth to 3 months; Diff.W6.W3: difference of weights at 3–6 months; ADWG.BW.W3; average daily weight gain from birth to 3 months; ADWG.W3.W6: average daily weight gain from 3 to 6 months. a,b: The means with the same letter in each row are not significantly different by Duncan’s multiple range test at 0.05 level. | | | | | | | | | | | | | | | |

**Effects of sex and mating systems on growth performance of lambs and fat-tail traits**

In the present study the female lambs have significantly lower growth rate compared to males (Table 4). Moreover, female lambs have greater FTH (18.10 cm vs. 15.48 cm) but lower FTW (15.53 cm vs. 18.12 cm) than male lambs (*P* < 0.05).

Fig 1 illustrates the use of four distinct mating systems (Fig 1, C, D, E, F) in producing crossbred lambs of F2 and BC1. Notably, the offspring resulting from paternal backcrosses exhibited significantly greater birth weights compared to those from the other mating systems (*P* < 0.0001, Table 5). While lambs born through pure-breeding, in-breeding, and outcrossing displayed higher average daily weight gain before weaning (ADWG.BW.W3) when contrasted with the backcrossed lambs, the situation shifted post-weaning. After weaning, the lambs born through backcrossing demonstrated a significantly higher growth rate (ADWG.W3.W6) compared to the others (*P* < 0.0001, Table 5). To enhance the growth rate, we have determined that paternal backcrossing is the optimal strategy for the introgression of major genes into the Moghani pure breed. When it comes to fat-tail traits, lambs born from pure-breeding Moghani sheep exhibited significantly greater FTH and FTW compared to lambs born from various mating systems, including in-breeding, outcrossing, paternal backcrossing, and maternal backcrossing (*P* < 0.0001, Table 5).

**Table 4. Predicted means for the effects of sex on lamb growth traits and fat-tail measurements in second generations of crossbred lambs (F2 and BC1)**

|  |  | **N** | **Lamb growth traits** | | | | | | | **Lamb fat-tail measurements** | |  |
| --- | --- | --- | --- | --- | --- | --- | --- | --- | --- | --- | --- | --- |
|  |  |  | **BW (kg)** | **W.3 (kg)** | **W.6 (kg)** | **Diff.W3.BW (kg)** | **Diff.W6.3 (kg)** | **ADWG.BW.W3 (gr)** | **ADWG.W3.W6 (gr)** | **FTH (cm)** | **FTW (cm)** |  |
| **Sex** | Male | 309 | 4.70 ^a^ | 27.50 ^a^ | 52.20 ^a^ | 22.94 ^a^ | 24.70 ^a^ | 254.90 ^a^ | 274.30 ^a^ | 15.48 ^b^ | 18.12 ^a^ |  |
|  | Female | 367 | 4.26 ^b^ | 24.95 ^b^ | 42.94 ^b^ | 20.73 ^b^ | 18.073 ^b^ | 230.30 ^b^ | 201 ^b^ | 18.10 ^a^ | 15.53 ^b^ |  |
| SEM | | | 0.04 | 0.17 | 0.39 | 0.16 | 0.33 | 1.81 | 3.69 | 0.32 | 0.28 |  |
| *P* Value | | | <0.0001 | <0.0001 | <0.0001 | <0.0001 | <0.0001 | <0.0001 | <0.0001 | <0.05 | <0.0001 |  |
| N=: number of lambs at each mating strategy; BW: birth weight; W.3: weight at 3 months (weaning); W.6: weight at 6 months; Diff.W3.BW: difference of weights at birth to 3 months; Diff.W6.W3: difference of weights at 3–6 months; ADWG.BW.W3; average daily weight gain from birth to 3 months; ADWG.W3.W6: average daily weight gain from 3 to 6 months; FTH: fat-tail height; FTW: fat-tail width. a,b: The means with the same letter in each part of each row were not significantly different in Duncan’s multiple range test at 0.05 level. | | | | | | | | | | | | |

**Table 5. Predicted means for the mating systems on lamb growth traits and fat-tail measurements of pure Moghani sheep and second generations of crossbred lambs (F2 and BC1)**

| **Mating strategy** | **N** | **Lamb growth traits** | | | | | | | **Fat-tail measurements** | |  |
| --- | --- | --- | --- | --- | --- | --- | --- | --- | --- | --- | --- |
|  |  | **BW (kg)** | **W.3 (kg) or weaning** | **W.6 (kg)** | **Diff.W3.BW (kg)** | **Diff.W6.3 (kg)** | **ADWG.BW.W3 (gr)** | **ADWG.W3.W6 (gr)** | **FTH (cm)** | **FTW (cm)** |  |
| **in-breeding** | 27 | 3.13 c | 24.89 ^bc^ | 37.77 ^d^ | 21.60 ^ab^ | 12.88 ^d^ | 240.00 ^ab^ | 143.10 ^d^ | 21.27 ^b^ | 1.57 ^d^ |  |
| **outcrossing** | 20 | 4.00 b | 26.18 ^ab^ | 43.53 ^bc^ | 21.94 ^ab^ | 17.34 ^c^ | 243.74 ^ab^ | 192.69 ^c^ | 16.05 ^c^ | 0.85 ^d^ |  |
| **paternal backcrossing** | 425 | 4.93 a | 25.98 ^ab^ | 50.87 ^a^ | 21.26 ^b^ | 24.94 ^a^ | 236.24 ^b^ | 277.14 ^a^ | 12.59 ^c^ | 17.11 ^b^ |  |
| **maternal backcrossing** | 32 | 3.40 c | 23.93 ^c^ | 44.10 ^b^ | 20.53 ^b^ | 20.16 ^b^ | 228.13 ^b^ | 224.04 ^b^ | 11.66 ^c^ | 12.69 ^c^ |  |
| **pure-breeding** | 172 | 3.88 b | 27.02 ^a^ | 40.24 ^cd^ | 23.14 ^a^ | 13.10 ^d^ | 257.08 ^a^ | 145.50 ^d^ | 28.29 ^a^ | 19.53 ^a^ |  |
| SEM | | 0.04 | 0.17 | 0.39 | 0.16 | 0.33 | 1.81 | 3.69 | 0.32 | 0.28 |  |
| *P* Value | | <0.0001 | <0.0001 | <0.0001 | <0.0001 | <0.0001 | <0.0001 | <0.0001 | <0.0001 | <0.0001 |  |
| N=: number of lambs at each mating strategy; BW: birth weight; W.3: weight at 3 months (weaning); W.6: weight at 6 months; Diff.W3.BW: difference of weights at birth to 3 months; Diff.W6.W3: difference of weights at 3–6 months; ADWG.BW.W3; average daily weight gain from birth to 3 months; ADWG.W3.W6: average daily weight gain from 3 to 6 months; FTH: fat-tail height; FTW: fat-tail width. a,b: The means with the same letter in each part of each row were not significantly different in Duncan’s multiple range test at 0.05 level. | | | | | | | | | | | |

**Effects of progeny strains on growth performance of lambs and fat-tail traits**

In this study, strains resulting from paternal backcrossing, namely TTMM-2, TDMM-2, BMMM-2, and BRMM-2, exhibited greater birth weight (BW), as well as W3 and W6 measurements, and growth rates when compared to other sheep strains (*P* < 0.05, Table 6). Conversely, strains derived from maternal backcrossing systems, such as MBRM-2 and MBMM-2, displayed lower body weight compared to pure Moghani sheep before weaning. However, after weaning, they exhibited significantly higher growth rates (*P* < 0.05, Table 6). Among the various strains assessed, TTMM-2 exhibited superior characteristics, including higher BW, W.3, W.6, Diff.W6.3, and ADWG.W3.W6 in comparison to other strains. However, Moghani pure sheep displayed greater Diff.W3.BW and ADWG.BW.W3 than TTMM-2, although the difference was not statistically significant (refer to Table 6). TTMM-2 originated from the backcrossing of Texel Tamlet×Moghani (TTM) rams with Moghani pure ewes. It's worth noting that the Texel lines of Tamlet and Dalzell are associated with MyoMAX and double muscling phenotypes in New Zealand Texel sheep, both of which lead to hyperplasia or an increase in muscle fiber count [14]. Despite the BRMBRM-2, BMMBMM-2, TTMTTM-2, and TDMTDM-2 sheep strains, resulting from inbreeding systems among F1 crossbred lambs, displaying lower birth weights, the Texel sheep strains, including TTMTTM-2 and TDMTDM-2, demonstrated significantly higher W3 and ADWG.BW.3 when compared to other sheep strains (*P* < 0.05, see Table 6). These findings suggest that these inbred Texel sheep strains perform better than other sheep strains in terms of reaching the weaning stage.

Regarding fat-tail measurements, BMMBMM-2, TTMTTM-2, TDMTDM-2, and TTMTDM-2, exhibited distinct tail phenotypes compared to other strains. Moghani pure sheep, on the other hand, had significantly higher FTH in comparison to the other strains (*P* < 0.05, as shown in Table 6). As for FTW, no significant difference was observed between Moghani pure sheep and the strains resulting from paternal backcrossing, including TDMM-2, TTMM-2, BMMM-2, and BRMM-2 (refer to Table 6).

**Table 6. Predicted means for the produced strains of progenies on growth traits and fat-tail measurements between pure Moghani sheep and second generations of crossbred lambs (F2 and BC1)**

| **Breed** | **Strains** | **N** | **Lamb growth traits** | | | | | | | **Fat-tail measurements** | |
| --- | --- | --- | --- | --- | --- | --- | --- | --- | --- | --- | --- |
|  |  |  | **BW (kg)** | **W.3 (kg) or weaning** | **W.6 (kg)** | **Diff.W3.BW (kg)** | **Diff.W6.3 (kg)** | **ADWG.BW.W3 (gr)** | **ADWG.W3.W6 (gr)** | **FTH (cm)** | **FTW (cm)** |
| **Booroola crossbred lambs** | **BRMBRM-2** | 17 | 2.72 ^d^ | 20.95 ^e^ | 36.45 ^ef^ | 18.02 ^d^ | 15.49 ^dc^ | 200.15 ^d^ | 172.13 ^cd^ | 21.25 ^bc^ | 3.14 ^d^ |
|  | **BMMBMM-2** | 4 | 2.70 ^d^ | 22.24 ^de^ | 31.17 ^f^ | 19.54 ^cd^ | 8.94 ^e^ | 217.05 ^cd^ | 99.3 ^e^ | 25 ^ab^ | 0 ^d^ |
|  | **BMMM-2** | 96 | 4.93 ^a^ | 26.10 ^abc^ | 50.73 ^ab^ | 21.23 ^abc^ | 24.67 ^ab^ | 235.84 ^bc^ | 274.12 ^ab^ | 13.15 ^d^ | 17.89 ^a^ |
|  | **BRMM-2** | 153 | 4.9 ^a^ | 26.40 ^abc^ | 52.23 ^a^ | 21.65 ^abc^ | 25.83 ^a^ | 240.60 ^abc^ | 287 ^a^ | 12.97 ^d^ | 16.38 ^ab^ |
|  | **MBRM-2** | 15 | 3.34 ^c^ | 23.80 ^cde^ | 44.72 ^bcd^ | 20.44 ^bcd^ | 20.94 ^ab^ | 227.06 ^bcd^ | 232.65 ^ab^ | 12.47 ^d^ | 12.47 ^c^ |
|  | **MBMM-2** | 17 | 3.46 ^c^ | 24.10 ^bcde^ | 43.55 ^bcde^ | 20.62 ^bcd^ | 19.48 ^bc^ | 229.06 ^bcd^ | 216.43 ^bc^ | 10.94 ^d^ | 12.88 ^bc^ |
| **Texel crossbred lambs** | **TTMTTM-2** | 8 | 3.75 ^bc^ | 27.81 ^a^ | 42.77 ^cde^ | 24.03 ^a^ | 14.95 ^dc^ | 267 ^a^ | 166.14 ^cd^ | 17 ^cd^ | 0 ^d^ |
|  | **TDMTDM-2** | 9 | 3.50 ^c^ | 27.76 ^a^ | 37.95 ^def^ | 24.26 ^a^ | 10.2 ^de^ | 269.5 ^a^ | 113.31 ^de^ | 20.5 ^bc^ | 0 ^d^ |
|  | **TDMTTM-2** | 14 | 4.23 ^b^ | 27.37 ^ab^ | 48.58 ^abc^ | 22.95 ^ab^ | 21.21 ^ab^ | 255 ^ab^ | 235.6 ^ab^ | 16.64 ^cd^ | 1.55 ^d^ |
|  | **TTMTDM-2** | 12 | 3.72 ^bc^ | 24.73 ^abcd^ | 37.35 ^ef^ | 20.7 ^bcd^ | 12.62 ^de^ | 229.98 ^bcd^ | 140.24 ^de^ | 15.33 ^cd^ | 0 ^d^ |
|  | **TDMM-2** | 96 | 4.80 ^a^ | 24.52 ^abcd^ | 47.69 ^abc^ | 20.10 ^bcd^ | 23.39 ^ab^ | 223.38 ^bcd^ | 259.88 ^ab^ | 11.59 ^d^ | 16.57 ^ab^ |
|  | **TTMM-2** | 63 | 5.22 ^a^ | 27.16 ^abc^ | 52.82 ^a^ | 22.20 ^abc^ | 25.67 ^a^ | 246.45 ^abc^ | 285.2 ^a^ | 12.39 ^d^ | 18.58 ^a^ |
| **Pure breed** | **Moghani** | 172 | 3.88 ^bc^ | 27.02 ^abc^ | 40.24 ^de^ | 23.14 ^ab^ | 13.1 ^de^ | 257.08 ^ab^ | 145.5 ^de^ | 28.28 ^a^ | 19.53 ^a^ |
| SEM | | | 0.04 | 0.17 | 0.39 | 0.16 | 0.33 | 1.81 | 3.69 | 0.32 | 0.28 |
| *P* Value | | | 0.0498 | 0.0125 | 0.0420 | 0.0089 | 0.04 | 0.0089 | 0.04 | 0.04 | 0.04 |
| N=: number of lambs at each strain; BW: birth weight; W.3: weight at 3 months (weaning); W.6: weight at 6 months; Diff.W3.BW: difference of weights at birth to 3 months; Diff.W6.W3: difference of weights at 3–6 months; ADWG.BW.W3; average daily weight gain from birth to 3 months; ADWG.W3.W6: average daily weight gain from 3 to 6 months; FTH: fat-tail height; FTW: fat-tail width. a,b: The means with the same letter in each part of each row were not significantly different in Duncan’s multiple range test at 0.05 level. | | | | | | | | | | | |

**Association of introgressed genes with growth traits**

Comparing the results of different genotypes of *MSTN* mutation (OAR2:g.118150665G>A) and *BMPR1B*/*Booroola* mutation (OAR6:g.29382188A>G) on growth traits, it was observed that homozygous reference and heterozygous genotypes for both genes significantly exhibited higher BW compared to the homozygous variant genotype and Moghani pure sheep (*P* < 0.05, as displayed in Table 7). Furthermore, as shown in Table 7, Moghani pure lambs were born heavier than F2 crossbred lambs with the *MSTN* mutation in the homozygous variant genotype (A/A), though the difference was not significant. In contrast, Moghani pure lambs were significantly (*P* < 0.05) heavier at birth compared to F2 crossbred lambs with the *Booroola* mutation in the homozygous variant genotype (G/G). This aligns with the findings of Çelikeloglu et al. [22] and Tekerli et al. [23], who conducted studies on the introgression of the *MSTN* mutation into Turkish Ramlıç sheep. Their findings indicated that Texel sheep with the *MSTN* mutation in homozygous form (A/A) exhibited significantly lower body weights in comparison to Ramlıç sheep with the wild-type genotype (G/G), as well as to the first-generation backcrosses (BC1, G/G and G/A) and the second-generation backcrosses (BC2, G/G and G/A) lambs [22,23]. Moreover, the A allele of the *MSTN* mutation OAR2:g.118150665G>A exerted a non-significant adverse impact on live weight traits in Texel sheep [57], Norwegian White sheep [58], Turkish Ramlıç sheep [22], and New Zealand Romney sheep [59]. Nevertheless, these studies did report a statistically significant positive effect on carcass and meat quality traits.

The significant interactions observed between subject effects, specifically strain × genotype and mating system × genotype (*P* < 0.0001), and suggest that birth weight in lambs is influenced by complex genetic and mating factors. The lower birth weight in homozygous variant genotypes could be attributed to the historical in-breeding and outcrossing practices, indicating that genetic diversity plays a crucial role in determining birth weight outcomes [60,61]. These findings highlight the importance of managing genetic diversity in breeding programs to improve lamb birth weight outcomes. Interestingly, the *MSTN* mutation in the homozygous variant genotype exhibited significantly higher values for W3, Diff.W3.BW, and ADWG.BW.W3 compared to other genotypes and pure Moghani sheep (*P* < 0.05, as shown in Table 7). Our findings align with other studies that have reported significant effects of the *MSTN* mutation OAR2:g.118150665G>A on the growth rate to weaning in New Zealand Romney [62] and Colored Polish Merino Sheep [63]. These results indicate that the *MSTN* mutation OAR2:g.118150665G>A in the homozygous variant genotype has a significant impact on lamb growth until weaning, which is crucial for sheep fattening programs and ewe reproduction management.

**Table 7. Predicted means for the genotype of introgressed gene on lamb growth traits between pure Moghani sheep and second generations of crossbred lambs (F2 and BC1)**

| **Introgressed gene** | **Genotype** | | **N** | **Lamb growth traits** | | | | | | |
| --- | --- | --- | --- | --- | --- | --- | --- | --- | --- | --- |
|  |  |  |  | **BW (kg)** | **W.3 (kg) or weaning** | **W.6 (kg)** | **Diff.W3.BW (kg)** | **Diff.W6.3 (kg)** | **ADWG.BW.W3 (gr)** | **ADWG.W3.W6 (gr)** |
| ***MSTN*** | homozygous reference | G/G | 103 | 4.77 a | 25.66 b | 49.65 a | 21.07 b | 24.23 a | 234.11 b | 269.22 a |
|  | heterozygous | G/A | 94 | 4.74 a | 25.72 b | 47.04 ab | 21.24 b | 21.32 ab | 236.00 b | 236.89 ab |
|  | homozygous variant | A/A | 6 | 3.63 b | 30.40 a | 47.11 ab | 26.36 a | 16.75 bc | 292.90 a | 186.13 bc |
| ***Booroola*** | homozygous reference | A/A | 139 | 4.58 a | 25.62 b | 49.68 a | 21.06 b | 24.06 a | 234.03 b | 267.35 a |
|  | heterozygous | A/G | 158 | 4.68 a | 26.03 b | 50.83 a | 21.44 b | 24.80 a | 238.16 b | 275.53 a |
|  | homozygous variant | G/G | 4 | 2.56 c | 23.96 b | 39.67 c | 21.45 b | 15.71 c | 238.40 b | 174.53 c |
| **Pure breed** | Moghani wild type | | 172 | 3.88 b | 27.02 b | 40.23 bc | 23.14 b | 13.10 c | 257.08 b | 145.50 c |
| SEM | | | | 0.04 | 0.17 | 0.39 | 0.16 | 0.33 | 1.81 | 3.69 |
| *P* Value | | | | 0.0031 | 0.0187 | 0.0144 | 0.04 | 0.0144 | 0.04 | 0.0144 |
| N=: number of lambs at each mating strategy; BW: birth weight; W.3: weight at 3 months (weaning); W.6: weight at 6 months; Diff.W3.BW: difference of weights at birth to 3 months; Diff.W6.W3: difference of weights at 3–6 months; ADWG.BW.W3; average daily weight gain from birth to 3 months; ADWG.W3.W6: average daily weight gain from 3 to 6 months. a,b: The means with the same letter in each part of each row were not significantly different in Duncan’s multiple range test at 0.05 level. | | | | | | | | | | |

In this study we provided additional confirmation that the *Booroola*/*FecB* does not adversely impact the growth rate, consistent with the findings reported in Garole × Malpura sheep [56]. Even in the homozygous variant state, no significant differences were found for Booroola lambs in the genotypes of homozygous reference, heterozygous, and homozygous variant when compared to Moghani pure sheep for the traits of W3, Diff.W3.BW, and ADWG.BW.W3 (*P* > 0.05, as shown in Table 7). We did not explore the impact of the *BMPR1B*/*Booroola* mutation on the prolificacy of crossbred lambs. Despite our thorough examination, prolificacy assessment awaits more data in future investigations. Nonetheless, previous studies have suggested that possessing one copy of the *Booroola* mutation (OAR_v.3.1; Chr 6: g.29382188A/G) leads to an increase in ovulation rate by 1.65 ova and in litter size by 0.9 lambs per lambing. Additionally, ewes that are homozygous (OAR_v.3.1; Chr 6: g.29382188G/G) for this mutation are estimated to experience additional increases of 1.65 ova shed and 0.3 lambs born per lambing (Davis, 2005; Gootwine, 2020). Importantly, Gootwine et al. [27] observed that ewes homozygous for this mutation demonstrate detrimental effects on lamb birth weight, post-weaning growth rate, and mature body weight. It has been underscored that, following the introduction of the *Booroola* mutation, breeding homozygous ewes is not recommended in commercial flocks due to significant lamb losses, despite the exceptionally high prolificacy observed in ewes with this genotype [24].

**Conclusions**

This study focused on examining how mating systems influence the performance of Moghani crossbred lambs, which are a mix of the Iranian indigenous Moghani breed and New Zealand sheep strains, including Texel and Booroola sheep. Among the mating systems employed in the development of second-generation crossbred lambs, both in-breeding and outcrossing consistently produced offspring with lean tails and woolly fleeces, emphasizing the heritability of these desirable traits. However, the examination of backcrossing progeny revealed a spectrum of tail phenotypes, ranging from short to large fat-tails, along with variations in fleece texture. We demonstrated that the utilization of paternal backcrossing emerges as a pivotal strategy for improving the growth rate and overall genetic potential of the Moghani pure breed. The deliberate choice to introgress major genes of *MSTN* and *Booroola* through this method underscores a commitment to precision and efficiency in breeding practices. The observed higher values for growth-related parameters in the *MSTN* mutation homozygous variant genotype indicate a lasting impact on lamb growth until weaning. This finding has significant implications for sheep fattening programs and ewe reproduction management within the Moghani sheep breed. By leveraging the advantages inherent in paternal backcrossing, specifically Texel Tamlet ram strains carrying the *MSTN* mutation, breeders can strategically enhance desirable traits within the Moghani breed, contributing to its resilience, adaptability, and productivity. The study emphasizes the importance of managing genetic diversity and considering both genetic markers and mating systems in sheep breeding programs.

**Acknowledgments**

The authors express their heartfelt appreciation to Jovain Agricultural & Industrial Company for supplying valuable data. Gratitude is extended to Mr. Hamid Jafar-Abadi, Mr. Mohammad Reza Ghale-Noei, Mr. Valiollah Annabestani, and the diligent animal husbandry team for their collaborative contributions to data collection and blood sampling.

**References**

1.	Leymaster KA, Jenkins TG. Comparison of Texel- and Suffolk-sired crossbred lambs for survival, growth, and compositional traits. J Anim Sci. 1993;71: 859–869. doi:10.2527/1993.714859x

2.	Notter DR. The importance of genetic diversity in livestock populations of the future. J Anim Sci. 1999;77: 61–69.

3.	Leymaster KA. Fundamental aspects of crossbreeding of sheep: use of breed diversity to improve efficiency of meat production. Sheep Goat Res J. 2002;17: 50–59.

4.	Talebi R, Ghaffari MR, Fabre S, Qanbari S. Comparison of the growth performance traits in F1 crossbred lambs between two strains Booroola Merino × Moghani and Booroola Romney × Moghani. 12th National And 4th International Biotechnology Congress of Islamic Republic of Iran. 2021. pp. 1–11.

5.	Talebi R, Ghaffari MR, Fabre S, Mardi M, Kazemi Alamouti M. Comparison of the growth performance between pure Moghani sheep and crosses with Texel or Booroola sheep carrying major genes contributing to muscularity and prolificacy. Anim Biotechnol. 2023;0: 1–12. doi:10.1080/10495398.2023.2165933

6.	Abebe A, Berhane G, Gizaw S, Getachew T, Haile A. Effect of genotype and environment on the productive and survivability traits of lambs under a community-based management system. J Agric Food Res. 2023;13: 100644. doi:10.1016/J.JAFR.2023.100644

7.	Abegaz S, Van Wyk JB, Olivier JJ. Estimation of genetic and phenotypic parameters of growth curve and their relationship with early growth and productivity in Horro sheep. Arch Anim Breed. 2010;53: 85–94. doi:10.5194/aab-53-85-2010

8.	Hopkins DL, Fogarty NM, Mortimer SI. Genetic related effects on sheep meat quality. Small Rumin Res. 2011;101: 160–172. doi:10.1016/j.smallrumres.2011.09.036

9.	Gootwine E. Invited review: Opportunities for genetic improvement toward higher prolificacy in sheep. Small Rumin Res. 2020;186: 106090. doi:10.1016/j.smallrumres.2020.106090

10.	Posbergh CJ, Huson HJ. All sheeps and sizes: a genetic investigation of mature body size across sheep breeds reveals a polygenic nature. Anim Genet. 2021;52: 99–107. doi:10.1111/age.13016

11.	Talebi R, Ahmadi A, Hajiloei Z, Ghaffari MR, Zeinalabedini M, Saki AA, et al. Association of ovine follistatin gene polymorphisms with body measurements , fat-tail traits and morphometric of head in Iranian Mehraban sheep. Small Rumin Res. 2023;225: 107020. doi:10.1016/j.smallrumres.2023.107020

12.	Kijas JW, Lenstra JA, Hayes B, Boitard S, Neto LR, Cristobal MS, et al. Genome-wide analysis of the world’s sheep breeds reveals high levels of historic mixture and strong recent selection. PLoS Biol. 2012;10. doi:10.1371/journal.pbio.1001258

13.	Bolormaa S, Swan AA, Brown DJ, Hatcher S, Moghaddar N, Van Der Werf JH, et al. Multiple-trait QTL mapping and genomic prediction for wool traits in sheep. Genet Sel Evol. 2017;49: 1–22. doi:10.1186/s12711-017-0337-y

14.	Talebi R, Ghaffari MR, Zeinalabedini M, Abdoli R, Mardi M. Genetic basis of muscle-related traits in sheep: A review. Anim Genet. 2022;53: 723–739. doi:10.1111/age.13266

15.	Broad TE, Glass BC, Greer GJ, Robertson TM, Bain WE, Lord EA, et al. Search for a locus near to myostatin that increases muscling in Texel sheep in New Zealand. 2000;60: 110–112. doi:10.13140/2.1.4980.6721

16.	Johnson PL, Dodds KG, Bain WE, Greer GJ, McLean NJ, McLaren RJ, et al. Investigations into the GDF8 g+6723G-A polymorphism in New Zealand Texel sheep. J Anim Sci. 2009;87: 1856–1864. doi:10.2527/jas.2008-1508

17.	Marshall K, Henshall J, Banks R, Werf J van der. Finding major gene effects in Australian meat sheep : feasibility study for a Texel dataset. Proc Assoc Adv Anim Breed Genet. 1999;13: 86–89.

18.	Clop A, Marcq F, Takeda H, Pirottin D, Tordoir X, Bibé B, et al. A mutation creating a potential illegitimate microRNA target site in the myostatin gene affects muscularity in sheep. Nat Genet. 2006;38: 813–818. doi:10.1038/ng1810

19.	Marcq F, Barkouki SE, Elsen J, Grobet L, Royo L, Leroy P, et al. Investigating the role of myostatin in the determinism of double muscling characterizing Belgian Texel sheep. undefined. 1998.

20.	Boman IA, Klemetsdal G, Blichfeldt T, Nafstad O, Våge DI. A frameshift mutation in the coding region of the myostatin gene (MSTN) affects carcass conformation and fatness in Norwegian White Sheep (Ovis aries). Anim Genet. 2009;40: 418–422. doi:10.1111/j.1365-2052.2009.01855.x

21.	Hadjipavlou G, Matika O, Clop A, Bishop SC. Two single nucleotide polymorphisms in the myostatin (GDF8) gene have significant association with muscle depth of commercial Charollais sheep. Anim Genet. 2008;39: 346–353. doi:10.1111/j.1365-2052.2008.01734.x

22.	Çelikeloglu K, Tekerli M, Eedogan M, Koçak S, Yazici E, Gücüyener Hacan Ö, et al. Marker-assisted introgression of myostatin from Texel to Ramlıç sheep: Growth and real-time ultrasound carcass traits in F1 and BC1 lambs. Ankara Üniversitesi Vet Fakültesi Derg. 2021; 25–31. doi:10.33988/auvfd.795247

23.	Tekerli M, Erdogan M, Koçak S, Çelikeloglu K, Yazıcı E, Hacan Ö, et al. The comparative results of myostatin introgression from donor Texel to recipient Ramlıç sheep with the aspects of growth, pre-, and post-slaughter carcass traits in the second backcross generation. Arch Anim Breed. 2022;65: 231–238. doi:10.5194/aab-65-231-2022

24.	Gootwine E. Invited review: Opportunities for genetic improvement toward higher prolificacy in sheep. Small Rumin Res. 2020;186: 106090. doi:10.1016/j.smallrumres.2020.106090

25.	Qanbari S, Osfoori R, Eskandari Nasab morad pasha. Marker Assisted Introgression of FecB gene into Afshari Sheep. Iran J Anim Sci. 2009;39: 39–47.

26.	Qanbari S, Osfoori R, Nasab MPE. A preliminary study of marker data applicability in gene introgression program for Afshari sheep breed. Biotechnology. Asian Network for Scientific Information; 2007. pp. 513–519. doi:10.3923/biotech.2007.513.519

27.	Gootwine E, Rozov A, Bor A, Reicher S. Carrying the FecB (Booroola) mutation is associated with lower birth weight and slower post-weaning growth rate for lambs, as well as a lighter mature bodyweight for ewes. Reprod Fertil Dev. 2006;18: 433–437. doi:10.1071/RD05134

28.	Gootwine E, Zenu A, Bor A, Yossafi S, Rosov A, Pollott GE. Genetic and economic analysis of introgression the B allele of the FecB (Booroola) gene into the Awassi and Assaf dairy breeds. Livest Prod Sci. 2001;71: 49–58. doi:10.1016/S0301-6226(01)00240-8

29.	Gootwine E, Reicher S, Rozov A. Prolificacy and lamb survival at birth in Awassi and Assaf sheep carrying the FecB (Booroola) mutation. Anim Reprod Sci. 2008;108: 402–411. doi:10.1016/j.anireprosci.2007.09.009

30.	Nimbkar C, Ghalsasi PM, Nimbkar BV, Ghalsasi PP, Gupta VS, Pardeshi VC, et al. Biological and economic consequences of introgression of the FecB (booroola) gene into deccani sheep. Proceedings of the International Booroola Workshop ACIAR Proceedings No 133 Australian Centre for International Agricultural Research, Pune, Maharashtra, India. 2009. pp. 90–99.

31.	Teyssier J, Bodin L, Maton C, Bouquet PM, Elsen JM. Biological and economic consequences of introgression of the FecB gene into the French Mérinos d’Arles sheep. In: Walkden-Brown, S.W., van der Werf, J.H.J., Nimbkar, C., Gupta VS, (Eds.) U of the F (Booroola) G in S-BP, editors. Proceedings of ACIAR Proceedinga No 133 Australian Centre for International Agricultural Research, Puna Maharashtra India. Helen Newton Turner Memorial International Workshop.; 2009. pp. 128–134.

32.	Esfandyari H, Aslaminejad AA, Rafat SA. Wool characteristics in the third generation of Arkharmerino × Ghezel and Arkharmerino × Moghani crossbreed sheep. Trop Anim Health Prod. 2011;43: 1337–1343. doi:10.1007/s11250-011-9862-9

33.	Mokhber Yousefabad M, Shodja J, Alijani S, Behruzlak M. Evaluation of Fleece Characterstics of First and Second Generations of ArkharMerinos×Ghezel and ArkharMerinos × Moghani Crossbred sheep. J Agric Sci Nat Resour. 2008;15: 149–156.

34.	Southey BR, Thomas DL, Gottfredson RG, Zelinsky RD. Ewe productivity of Booroola Merino-Rambouillet crossbred sheep during early stages of the introgression of the Fecb allele into a rambouillet population. Livest Prod Sci. 2002;75: 33–44. doi:10.1016/S0301-6226(01)00301-3

35.	Talebi R, Ahmadi A, Afraz F, Sarry J, Woloszyn F, Fabre S. Detection of single nucleotide polymorphisms at major prolificacy genes in the Mehraban sheep and association with litter size. Ann Anim Sci. 2018;18. doi:10.2478/aoas-2018-0014

36.	Majd SA, Ahmadi A, Talebi R, Koohi PM, Fabre S, Qanbari S. Polymorphism identification in ovine KISS1R/GPR54 gene among pure and crossbreeds of Iranian sheep. Small Rumin Res. 2019;173. doi:10.1016/j.smallrumres.2019.02.005

37.	Ahmadi A, Mohammadizade M, Zamani P, Farah-avar A, Talebi R. Identification of Polymorphism in HSP70A1A Gene in Mehraban and Romanov Sheep Breeds using PCR-SSCP Technique. Res Anim Prod Res. 2021;12: 169–179. Available: https://rap.sanru.ac.ir/article-1-1156-en.html

38.	Jasouri M, Aljani S, Talebi R, Hasanzadeh A. Influence of maternal effects on estimation of genetic parameters of growth traits in Ghezel sheep using bayesian via Gibbs sampling technique. J Anim Sci Res. 2014;24: 47–55.

39.	Bakhshalizadeh S, Hashemi A, Gaffari M, Jafari S, Farhadian M. Estimation of genetic parameters and genetic trends for biometric traits in Moghani sheep breed. Small Rumin Res. 2016;134: 79–83. doi:10.1016/j.smallrumres.2015.12.030

40.	Hossein-Zadeh NG. Modelling growth curve in Moghani sheep: Comparison of non-linear mixed growth models and estimation of genetic relationship between growth curve parameters. J Agric Sci. 2017;155: 1150–1159. doi:10.1017/S0021859617000326

41.	Jafaroghli M, Rashidi A, Mokhtari MS, Shadparvar AA. (Co)Variance components and genetic parameter estimates for growth traits in Moghani sheep. Small Rumin Res. 2010;91: 170–177. doi:10.1016/j.smallrumres.2010.03.010

42.	Rashidi A, Mokhtari MS, Esmailizadeh AK, Asadi Fozi M. Genetic analysis of ewe productivity traits in Moghani sheep. Small Rumin Res. 2011;96: 11–15. doi:10.1016/j.smallrumres.2010.11.001

43.	Talebi R, Seighalani R, Qanbari S. A handmade DNA extraction kit using laundry powder; insights on simplicity, cost-efficiency, rapidity, safety and the quality of purified DNA. Anim Biotechnol. 2019; 1–7. doi:10.1080/10495398.2019.1684933

44.	Statistical Analysis System (2001) User’s Guide: Statistics, Version 8.2. SAS Institute, NC, USA.

45.	Daetwyler HD, Villanueva B, Bijma P, Woolliams JA. Inbreeding in genome-wide selection. J Anim Breed Genet. 2007;124: 369–376. doi:10.1111/j.1439-0388.2007.00693.x

46.	Khaldari M, Azarfar A, Pahlavan R. The size of fat tail does not have an effect on growth performance and carcass characteristics in Lori-Bakhtiari lambs. Small Rumin Res. 2020;187: 106088. doi:10.1016/j.smallrumres.2020.106088

47.	Khaldari M, Kashan NEJ, Afzalzadeh A, Salehi A. Growth and carcass characteristics of crossbred progeny from lean-tailed and fat-tailed sheep breeds. S Afr J Anim Sci. 2007;37: 51–56.

48.	Kalds P, Luo Q, Sun K, Zhou S, Chen Y, Wang X. Trends towards revealing the genetic architecture of sheep tail patterning: Promising genes and investigatory pathways. Anim Genet. 2021;52: 799–812. doi:10.1111/age.13133

49.	Kalds P, Zhou S, Gao Y, Cai B, Huang S, Chen Y, et al. Genetics of the phenotypic evolution in sheep: a molecular look at diversity-driving genes. Genet Sel Evol. 2022;54: 1–27. doi:10.1186/s12711-022-00753-3

50.	Malik RC, Singh RN, Acharya RM, Dutta OP. Factors affecting lamb survival in crossbred sheep. Trop Anim Health Prod. 1980;12: 217–223. doi:10.1007/BF02236619

51.	Walkden-Brown SW, Wolfenden DH, Piper LR. Biological and economic consequences of introgression of the FecB mutation into merino sheep in Australia. In: In: Walkden-Brown, S.W., van der Werf, J.H.J., Nimbkar, C., Gupta VS (Eds. ., editor. Proceedings of Helen Newton Turner Memorial International Workshop ACIAR Procedings No 133. Australian Centre for International Agricultural Research, Pune Maharashtra India. Use Programs., of the FecB (Booroola) Gene in Sheep-Breeding; 2009. pp. 100–110.

52.	Notter DR. Genetic improvement of reproductive efficiency of sheep and goats. Anim Reprod Sci. 2012;130: 147–151. doi:10.1016/J.ANIREPROSCI.2012.01.008

53.	Moore LG, Charles SM, Julian CG. Humans at high altitude: Hypoxia and fetal growth. Respir Physiol Neurobiol. 2011;178: 181–190. doi:10.1016/J.RESP.2011.04.017

54.	Loerch SC, Mcclure KE, Parker CF. Effects of Number of Lambs Suckled and Supplemental Protein Source on Lactating Ewe Performance. J Anim Sci. 1985;60: 6–13. doi:10.2527/jas1985.6016

55.	McHugh N, Pabiou T, McDermott K, Wall E, Berry DP. Impact of birth and rearing type, as well as inaccuracy of recording, on pre-weaning lamb phenotypic and genetic merit for live weight. Transl Anim Sci. 2017;1: 137–145. doi:10.2527/tas2017.0015

56.	Kumar S, Mishra AK, Kolte AP, Arora AL, Singh D, Singh VK. Effects of the Booroola (FecB) genotypes on growth performance, ewe’s productivity efficiency and litter size in Garole × Malpura sheep. Anim Reprod Sci. 2008;105: 319–331. doi:10.1016/j.anireprosci.2007.03.012

57.	Johnson PL, McEwan JC, Dodds KG, Purchas RW, Blair HT. A directed search in the region of GDF8 for quantitative trait loci affecting carcass traits in Texel sheep. J Anim Sci. 2005;83: 1988–2000. doi:10.2527/2005.8391988x

58.	Boman IA, Klemetsdal G, Nafstad O, Blichfeldt T, Våge DI. Impact of two myostatin (MSTN) mutations on weight gain and lamb carcass classification in Norwegian White Sheep (Ovis aries). Genet Sel Evol. 2010;42: 1–7. doi:10.1186/1297-9686-42-4

59.	Hickford JGH, Forrest RH, Zhou H, Fang Q, Han J, Frampton CM, et al. Polymorphisms in the ovine myostatin gene (MSTN) and their association with growth and carcass traits in New Zealand Romney sheep. Anim Genet. 2010;41: 64–72. doi:10.1111/j.1365-2052.2009.01965.x

60.	Rafter P, McHugh N, Pabiou T, Berry DP. Inbreeding trends and genetic diversity in purebred sheep populations. Animal. 2022;16: 100604. doi:10.1016/j.animal.2022.100604

61.	Leroy G. Inbreeding depression in livestock species: Review and meta-analysis. Anim Genet. 2014;45: 618–628. doi:10.1111/age.12178

62.	Han J, Forrest RH, Sedcole JR, Hickford JGH. Myostatin ( MSTN ) gene haplotypes and their association with growth and carcass traits in New Zealand Romney lambs. Small Rumin Res. 2015;127: 8–19. doi:10.1016/j.smallrumres.2015.03.015

63.	Grochowska E, Borys B, Mroczkowski S. Effects of intronic SNPs in the myostatin gene on growth and carcass traits in colored polish merino sheep. Genes (Basel). 2020;11: 1–18. doi:10.3390/genes11010002

64.	Davis G. Major genes affecting ovulation rate in sheep. Genet Sel Evol. 2005;37: S11. doi:10.1186/1297-9686-37-s1-s11
